# Interactive effects of inbreeding and endocrine disruption on reproduction in a model laboratory fish

**DOI:** 10.1111/j.1752-4571.2012.00288.x

**Published:** 2012-08-30

**Authors:** Lisa K Bickley, Andrew R Brown, David J Hosken, Patrick B Hamilton, Gareth Le Page, Gregory C Paull, Stewart F Owen, Charles R Tyler

**Affiliations:** 1 University of Exeter, Biosciences, College of Life and Environmental SciencesExeter, UK; 2 AstraZeneca Safety, Health and Environment, Brixham Environmental LaboratoryBrixham, UK; 3 University of Exeter, Biosciences, Centre for the Environment and ConservationCornwall, UK

**Keywords:** ecotoxicology, fitness, fungicide, zebrafish, reproduction

## Abstract

Inbreeding depression is expected to be more severe in stressful environments. However, the extent to which inbreeding affects the vulnerability of populations to environmental stressors, such as chemical exposure, remains unresolved. Here we report on the combined impacts of inbreeding and exposure to an endocrine disrupting chemical (the fungicide clotrimazole) on zebrafish (*Danio rerio*). We show that whilst inbreeding can negatively affect reproductive traits, not all traits are affected equally. Inbreeding depression frequently only became apparent when fish were additionally stressed by chemical exposure. Embryo viability was significantly reduced in inbred exposed fish and there was a tendency for inbred males to sire fewer offspring when in direct competition with outbred individuals. Levels of plasma 11-ketotestosterone, a key male sex hormone, showed substantial inbreeding depression that was unaffected by addition of the fungicide. In contrast, there was no effect of inbreeding or clotrimazole exposure on egg production. Overall, our data provide evidence that stress may amplify the effects of inbreeding on key reproductive traits, particularly those associated with male fitness. This may have important implications when considering the consequences of exposure to chemical pollutants on the fitness of wild populations.

## Introduction

As anthropogenic pressures on the environment increase, wildlife populations are becoming more fragmented. Smaller and more isolated populations are not only more vulnerable to external environmental changes and chance fluctuations in local survival (Keller and Waller 2002) but inbreeding, or mating between closely related individuals, may become more prevalent (Höglund 2009).

One significant stressor of wildlife populations is exposure to chemicals discharged as a consequence of anthropogenic activities. The presence of novel, man-made chemicals in the environment is a relatively new pressure faced by wildlife populations, with potential evolutionary implications. Environmental perturbations such as toxicant exposure may lead to particularly strong novel selection pressures, which have the potential to cause the evolution of heritable traits over relatively few generations (contemporary evolution) (Stockwell et al. 2003). Some species or populations have been shown to adapt to environmental disturbances (for example, mosquitofish (*Gambusia affinis,* Baird and Girard) exposed to pesticides (Andreasen 1985), killifish (*Fundulus heteroclitus,* Linnaeus) exposed to various hydrocarbons (Nacci et al. 1999), and tomcod (*Microgadus tomcod*, Walbaum) exposed to polychlorinated biphenyls (PCBs) (Wirgin et al. 2011), and genome scans have shown that adaptation to such habitats can involve multiple genomic regions (Williams and Oleksiak 2008). However, most forms of adaptation result in a selective loss of genetic variation as the frequency of genotypes that improve fitness increase (Stockwell et al. 2003). Furthermore, small populations are often considered to have limited potential for such adaptive evolution because of reduced genetic diversity (Franklin and Frankham 1998). Therefore, reductions in population size as well as the increased isolation of populations through habitat degradation are not only likely to impact on inbreeding but also on the complex interactions between inbreeding, reduced genetic diversity and the ability of populations to respond to current and future environmental disturbances.

The vast array of chemicals present in the environment means wildlife may be exposed to multiple chemicals with the potential to cause adverse effects via various physiological routes. One particular group causing widespread concern are endocrine disrupting chemicals (EDCs). These affect the hormonal systems of animals through both receptor- and non-receptor-mediated pathways, and exposure to EDCs is associated with deleterious impacts on reproduction and other aspects of health in a wide range of wildlife taxa, including invertebrates, fish, amphibians, reptiles, birds and mammals (reviewed in Tyler and Goodhead 2010). Exposure to some EDCs may also negatively impact on population dynamics in wildlife (Bryan et al. 1986; Fry 1995; Kidd et al. 2007; Harris et al. 2011) and as a consequence, considerable efforts are being directed at testing chemicals for endocrine disrupting properties.

Chemical toxicity testing in mammals typically employs inbred isogenic laboratory animals. This is because they show less phenotypic variation (NAS 2007), thereby reducing the numbers of animals needed in testing programs. Many of the laboratory strains used in ecotoxicological evaluations tend to be less genetically diverse than their wild counterparts (Woods et al. 1989; Nowak et al. 2007; including zebrafish (*Danio rerio*, Hamilton), Coe et al. 2009; Whiteley et al. 2011), although this is rarely quantified or reported. However, a fundamental aim of ecotoxicity testing is to protect and prevent adverse effects in wild animals, and hence the use of outbred strains has been argued to be more appropriate, as they may better represent wild populations (Brown et al. 2009). The few reported studies on combined inbreeding and chemical exposure effects (all of which are on invertebrates) generally show that inbreeding increases the impacts of chemical exposure in laboratory-maintained animals. For example, inbred midges (*Chironomus riparius*, Meigen) showed a greater reduction in fitness when exposed to cadmium (Nowak et al. 2007), and inbreeding depression, a reduction in trait values because of inbreeding, was greater in the fruit fly (*Drosophila melanogaster*, Meigen) under environmental stress conditions, including exposure to DDT (Bijlsma et al. 1999). To our knowledge, there have been no reported investigations into the combined effects of inbreeding and chemical exposure on the reproduction of fish under controlled experimental conditions. Therefore, evidence to support the use of either inbred or more genetically variable outbred fish strains in ecotoxicological tests is currently lacking.

In addition to potential negative synergies between inbreeding and chemical exposure (or other stressors), inbreeding itself can also have negative fitness consequences because it can lead to inbreeding depression. Inbreeding depression has been documented for birth weight, survival, reproduction and resistance to environmental stressors (reviewed in Keller and Waller 2002). Many male characters closely related to fitness seem especially susceptible to inbreeding depression, presumably because of their history of directional selection (Michalczyk et al. 2010; Prokop et al. 2010; Okada et al. 2011). It might be predicted therefore that inbreeding depression for male traits linked to fitness is more severe under chemical stress (Meagher et al. 2000; Armbruster and Reed 2005). Again this has not been tested in fish.

Here, we investigated potential interactions between inbreeding and chemical exposure on the reproductive success of the zebrafish. The chosen chemical for this work, clotrimazole, is used in agriculture and in veterinary and human medicine. Clotrimazole is an imidazole fungicide and inhibits cytochrome P450 14α-lanosterol demethylase (CYP51; Lupetti et al. 2002). CYP51 is the most widely distributed P450 gene family, being found in animals, plants, fungi, yeast, lower eukaryotes and bacteria (Lepesheva and Waterman 2004). It plays a key role in cholesterol biosynthesis (Debeljak et al. 2003) and in the production of meiosis-activating sterols (Zarn et al. 2003). Azole compounds, like clotrimazole, have also been shown to inhibit several other P450 enzymes (Zhang et al. 2002), including aromatase (Zarn et al. 2003) an important enzyme involved in steroidogenesis and converting androgens to estrogens, and have been shown to disrupt steroidogenesis in fish (Ankley et al. 2007; Brown et al. 2011). Clotrimazole is designated as a priority hazardous substance by the European Union (OSPAR 2005).

We exposed inbred and outbred zebrafish to clotrimazole, and measures of reproductive output (number of eggs spawned and embryo viability) and competitive siring success were determined, the latter via paternity assessments of the offspring using DNA microsatellite genotyping. The key objectives were to determine whether exposure to an EDC affected reproductive output to a greater extent in inbred fish compared to outbred fish and whether inbred males were less reproductively successful compared to outbred males in competitive breeding trials. This information is not only important for a better understanding of the efficacy of ecotoxicology studies, but also for our understanding of how chemicals can impact endangered species, which, almost by definition, are found in small inbred populations. Furthermore, inbreeding potentially alters phenotypic and genetic variances (Falconer 1989). This in turn influences fitness landscapes and evolution, potentially facilitating population divergence (Whitlock 1995), especially when inbred and outbred populations respond differently to similar selection.

## Materials and methods

### Generation of inbred and outbred fish

**Figure 1:**
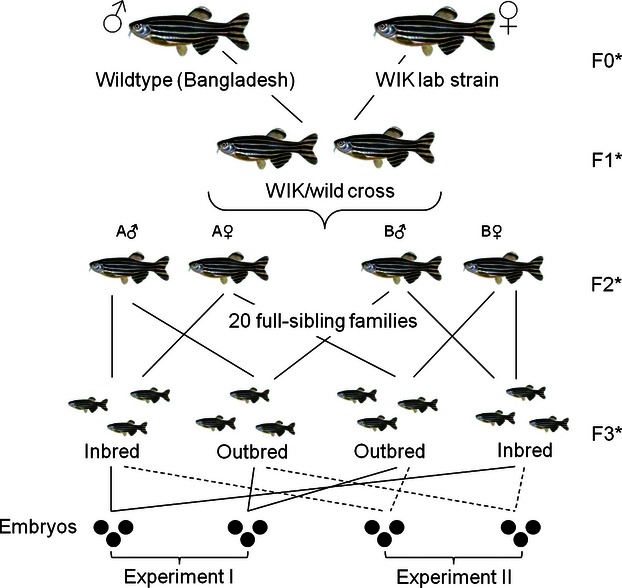
Generation of inbred and outbred zebrafish lines through controlled pairwise crosses. F0–F3* shows annotation corresponding to Brown et al. 2011.

The study was performed using replicate inbred and outbred lines generated through controlled zebrafish matings at the University of Exeter. Briefly, second-generation wild male zebrafish (of Bangladesh origin) were mated with Wild Indian Karyotype (WIK) females to generate hybrid WIK/wild strain fish (*F*_1_, Fig. 1), from which 20 full-sibling families were produced (*F*_2_, Fig. 1). From these, pairwise crosses were performed randomly to generate 20 outbred and 19 (one family failed to reproduce) inbred (via full-sibling mating) family lines (*F*_3_, Fig. 1). The theoretical inbreeding coefficients of F_3_ inbred and outbred fish used as parents in the breeding study were *F*_*IT*_ = *n* + 0.25 and *F*_*IT*_ = n, respectively, n representing the unknown inbreeding coefficients of WIK and wild zebrafish. Genetic variation at 12 microsatellite markers for both inbred and outbred zebrafish is reported in Brown et al. (2011).

### Exposure regime

At 37 days post-hatch (dph), F_3_ fish were either exposed to clotrimazole (CAS no. 23593-75-1) at a single nominal concentration of 5 μg L^−1^ or maintained in water without chemical treatment for 123 days (until 160 dph). Clotrimazole has a predicted environmental concentration (PEClocal) of 0.2 μg L^−1^ (OSPAR 2005) and higher concentrations (43.7 μg L^−1^) have been shown to cause significant (>90%) skew in sex ratio towards males (Brown et al. 2011). The concentration of clotrimazole was measured in the water throughout the study using tandem liquid chromatography and mass spectrometry as described in Brown et al. (2011), further details are reported in Table S1 of the supporting information. During the *in vivo* exposure, fish were maintained in 60 L (working volume) glass aquaria divided into eight separate compartments, each holding eight fish (of the same family group). Further details of the exposure regime can be found in Brown et al. (2011). Inbred and outbred fish were maintained separately, preventing any olfactory (pheromonal) contact. Details of the experimental conditions are provided (Table S1 of the supporting information).

**Figure 2:**
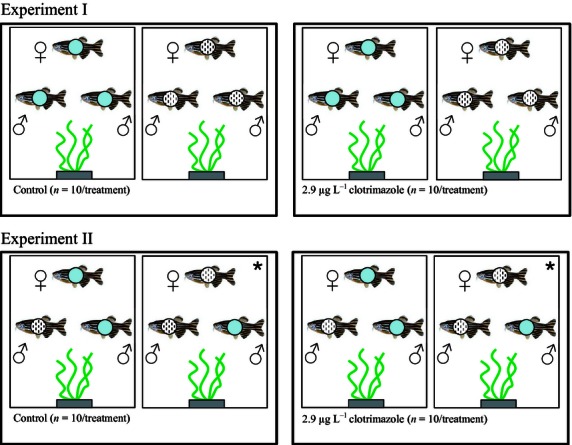
Experiment I: all fish in each spawning group were either inbred (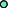) or outbred (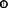) and had either been previously maintained under control conditions or exposed to clotrimazole; Experiment II: spawning groups contained one female (either inbred or outbred) and two males (one inbred and one outbred), and all fish in each spawning group had either been previously maintained under control conditions or exposed to clotrimazole. *denotes treatment used for subsequent parentage analysis.

Two experiments were conducted to investigate the combined effects of inbreeding and exposure to clotrimazole on the reproductive success of zebrafish. In the first experiment, spawning groups were established in which all fish in each group were either inbred or outbred (i.e. inbred and outbred fish did not directly compete) and had been either exposed to clotrimazole or maintained under control conditions (a fully factorial design, Fig. 2 Experiment I). Reproductive output was assessed in terms of number of eggs spawned and the proportion of live embryos. In the second experiment, the effect of clotrimazole exposure was investigated in a competitive breeding scenario, with inbred and outbred males directly competing for either an inbred or outbred female. As in the first experiment, all fish in each group had either been previously maintained under control conditions or exposed to clotrimazole (again a fully factorial design, Fig. 2 Experiment II). In addition to assessing reproductive output, paternity was also determined using DNA microsatellites. Because of cost constraints, we restricted our assessment to triads that contained an outbred female only, as previous (non-fish) studies indicate that male fitness is more susceptible to inbreeding (Saccheri et al. 1996).

### Experimental design I: reproductive output assessed in inbred and in outbred fish

At 160 dph, clotrimazole exposure was terminated and fish of breeding condition from all treatment groups were transferred into the breeding aquaria. Fish were maintained in control water conditions as described earlier (Table S1). Forty separate spawning groups were established (20 controls and 20 exposed; see Fig. 2 Experiment I) and each group, comprising one female and two males (either all inbred or all outbred), were randomly assigned to breeding aquaria. All fish within each spawning group were from different families, having previously been maintained in separate compartments and were sized matched using wet weights. The mean standard length, wet weight and average size difference between fish in each group are provided in Table S2, the supporting information.

The fish were acclimated to test conditions for 2 days before the experiment commenced. For 10 consecutive days, reproductive output was determined for all 40 spawning groups. Eggs were collected daily, 2–3 h after the dawn transition, by removing the spawning tray from each aquarium. The eggs were cleaned in aquarium water and counted, and unfertilized/fungal-infected eggs were discarded. Fertilized eggs were incubated in aerated aquarium water at 28 ± 1°C, and fertilization rates were measured at 2, 8 and 24 h post-fertilization (hpf).

### Experimental design II: reproductive output assessed incorporating direct competition between inbred and outbred males

Fish were allocated to 40 spawning groups (20 controls and 20 exposed), as described in Fig. 2 Experiment II. Each group contained one female (either inbred or outbred) and two males (one inbred and one outbred). As above, all fish within each spawning group were from different families and were sized matched using wet weights. The mean standard length, wet weight and average size difference between fish in each group are provided in Table S2. Reproductive output was recorded for 10 consecutive days, and fertilization rates measured at 2, 8 and 24 hpf as described earlier. Fertilized eggs were incubated until approximately 32 h prior to storage in 100% ethanol for subsequent paternity analysis.

At the end of the breeding studies, all fish were sacrificed by terminal anaesthesia (in 500 mg benzocaine, followed by destruction of the brain) in accordance with the UK Animals (Scientific Procedures) Act 1986. Fish were measured (standard length and wet weight) and a fin clip was taken and stored in 100% ethanol for subsequent paternity analysis. Blood was collected from each fish using heparinized capillary tubes, centrifuged and the plasma stored at −20°C for analysis of 11-ketotestosterone levels.

### 11-ketotestosterone quantification

11-Ketotestosterone (11-kt) is the most active androgen in male fish; it stimulates secondary sexual characteristics, spermatogenesis and reproductive behaviour, and the correlation between 11-kt concentrations, behavioural dominance and breeding performance has been widely documented in fish (reviewed in Borg 1994). 11-kt was quantified at the Centre for Environment, Fisheries and Aquaculture Science (CEFAS, Weymouth, UK) from 1-μL plasma using radioimmunoassay according to the method described by Scott et al. (1984). The detection limit of the assay was 1.22 ng 11-kt mL^−1^.

### Paternity analysis

Paternity analysis was conducted on twenty fertilized eggs randomly taken from each triad across the experimental period: the number of eggs sampled per day was in direct proportion to the total number of eggs spawned on that day. In total, 378 eggs were genotyped. All adult fish (*n* = 20 inbred males, 20 outbred males and 20 outbred females) were genotyped from fin clips collected at the termination of the study.

DNA was extracted from parental fins and fertilized eggs using ammonium acetate precipitation (adapted from Bruford et al. 1998). Six microsatellite loci [Z249, Z6104, Z9230, Z20450, Z4830 (http://www.zfin.org) and Ztri1 (Coe et al. 2008)] were used to assign paternity following Coe et al. (2008). 98.5% of all embryos tested could be assigned to a single parental pair. Fertilized eggs unable to be unequivocally assigned to sires were not included in the analyses.

### Data analysis

All statistical analyses were carried out using SigmaStat 3.1 (Systat Software Inc., Chicago, IL, USA) unless otherwise stated. Throughout the article, data are presented as mean ± one standard error of the mean. Data were assessed for normality and homogeneity of variances using the Kolmogorov–Smirnov and Levene's median tests, respectively. If parametric assumptions were met, data were analysed using analysis of variance (anova) with *post hoc* assessment of differences made using the Holm–Sidak multiple comparison method. When normality assumptions were not met, Kruskal–Wallis one-way anova was used to compare medians and *post hoc* Dunn's multiple comparison procedure applied as appropriate. Data for proportion of offspring sired by inbred and outbred males were analysed using a generalized linear model (GLM), with binomial errors and permutation tests (100 000), using the software R 2.8.1 (R: A Language and Environment for Statistical Computing, R Core Team, Vienna, Austria). Inbreeding depression was estimated for a number of traits (embryo viability, number of eggs produced, siring success and plasma levels of 11-kt) by calculating the coefficient of inbreeding depression (δ):


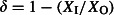


where *X*_I_ is the mean trait value for inbred progeny and *X*_O_ is the mean trait value for outbred progeny. Positive δ values indicate that trait values of outbreds exceed those of inbreds, whereas negative δ values indicate the opposite.

## Results

### Clotrimazole exposure concentrations

The geometric mean measured clotrimazole concentration was 2.9 μg L^−1^ (58% of the nominal 5 μg L^−1^; Table S1).

### Experiment I: reproductive output assessed in inbred and in outbred fish

#### Reproductive output

**Figure 3:**
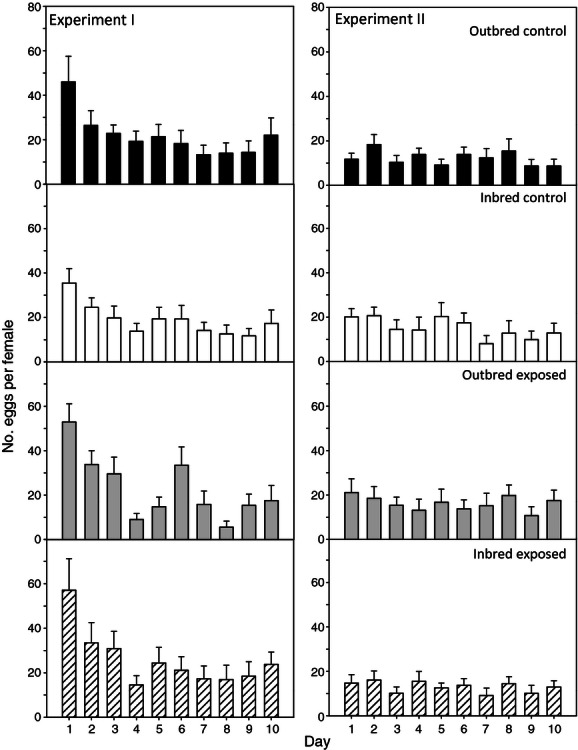
The effect of inbreeding and clotrimazole exposure on number of eggs spawned per female, over the 10-day study period of Experiment (I) and Experiment (II). Data presented are mean ± SE, *n* = 10. Each spawning group contained one female and two males. In Experiment I, all fish were either outbred or inbred and had either been previously maintained under control conditions or exposed to clotrimazole, as indicated. In Experiment II each spawning group contained one female (either inbred or outbred, as indicated) and two males (one inbred and one outbred), and all fish had either been previously maintained under control conditions or exposed to clotrimazole, again as indicated.

**Figure 4:**
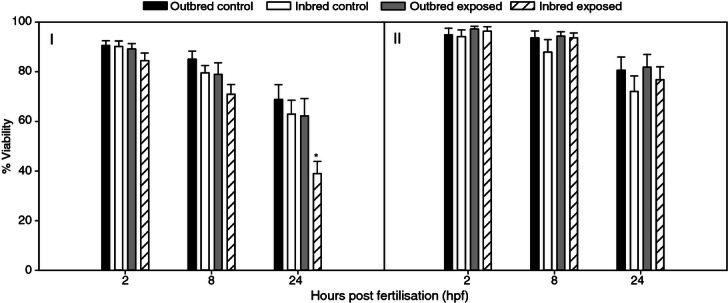
The effect of clotrimazole exposure on embryo viability at 2, 8 and 24 h post-fertilization (hpf), across the 10-day study period of Experiment (I) and Experiment (II). Data presented are mean ± SE, *n* = 10. * denotes significantly different to all other treatment groups (*P* = <0.001). In Experiment I, all fish were either outbred or inbred and had either been previously maintained under control conditions or exposed to clotrimazole, as indicated. In Experiment II each spawning group contained one female (either inbred or outbred, as indicated) and two males (one inbred and one outbred), and all fish had either been previously maintained under control conditions or exposed to clotrimazole, again as indicated.

The mean numbers of eggs produced per spawning group per day (batch size) were as follows: control outbred fish, 26 ± 3; control inbred fish, 24 ± 2; exposed outbred fish, 26 ± 4, and exposed inbred fish, 27 ± 4 (Fig. 3 Experiment I). Whilst there was variation in the number of eggs produced on each study day (two-way repeated measures analysis of variance (anova), *F*_9,324_ = 17.23, *P* = <0.001), there was no overall difference in the number of eggs spawned between inbred and outbred females (*P* = 0.992), exposed or non-exposed (*P* = 0.336), and there was no statistical interaction between study day and treatment groups (*P* = 0.470). Embryo viability declined across all treatment groups over the 24 h period after egg collection (two-way anova with Holm–Sidak multiple comparison procedure, *F*_2,108_ = 52.0, *P* = <0.001, Fig. 4 Experiment I). However, at 24 hpf, viability was significantly reduced in inbred fish exposed to clotrimazole (39.0 ± 4.9%) compared to all other treatment groups (68.8 ± 6.0%, 62.9 ± 5.6% and 62.2 ± 7.1% in outbred control, inbred control and outbred exposed, respectively) (*F*_3,108_ = 8.44, *P* = <0.001, Fig. 4 Experiment I). There was no statistically significant interaction between different treatment groups and the time of analysis (*F*_6,108_ = 1.6, *P* = 0.151).

### Experiment II: reproductive output assessed incorporating direct competition between inbred and outbred males

#### Reproductive output

The mean number of eggs produced per spawning group per day was as follows: control fish with outbred female, 12 ± 4; control fish with inbred female, 15 ± 1; exposed fish with outbred female, 16 ± 2; and exposed fish with inbred female, 13 ± 1. Similar to Experiment I, the daily number of eggs produced showed significant variation across the experimental period (two-way repeated measures anova, *F*_9,324_ = 2.62, *P* = 0.006; Fig. 3 Experiment II), but no differences were seen between inbred and outbred (*P* = 0.948), or exposed and non-exposed females (*P* = 0.766), and there was no statistical interaction between study day and treatment groups (*P* = 0.309). In this experiment, whilst there was a decline in embryo viability across all treatment groups over time (two-way anova with Holm–Sidak multiple comparison procedure, *F*_2,107_ = 24.9, *P* = <0.001, Fig. 4 Experiment II), there was no significant differences between treatment groups (*F*_3,107_ = 1.61, *P* = 0.191; Fig. 4 Experiment II). There was also no statistically significant interaction between treatment groups and time of analysis (*F*_6,107_ = 0.35, *P* = 0.91). Embryo viability at 24 hpf in each treatment group was as follows: 89.6% ± 2.2 for outbred control, 91.1% ± 2.2 for outbred exposed, 84.7% ± 2.5 for inbred control and 89.8% ± 2.2 for inbred exposed, respectively. The lack of significant differences in embryo viability (which were seen in Experiment I) could be because outbred males sired more offspring in the fertilization competition between inbred and outbred males (see Paternity analysis and 11-kt) and therefore ‘rescued’ embryo viability.

#### Paternity analysis

**Figure 5:**
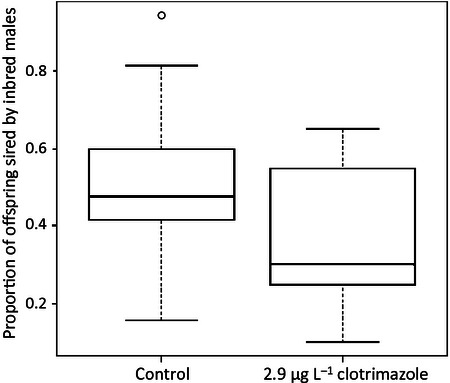
Proportion of offspring sired by inbred males in competition with outbred males in control and exposed treatment groups (GLM with binomial error; *F* = 0.6221; df = 1, 18; *P* = 0.058). Data analysed for spawning groups containing outbred females only. The central line within the box is the median. The box represents the upper and lower quartiles, and the whiskers the 95% confidence intervals. Open circles are outliers beyond the 95% confidence intervals. *n* = 9.

Paternity analysis revealed that in non-exposed triads, outbred males sired on average 47% (±0.07) of the offspring (Fig. 5). Following exposure to clotrimazole, this proportion increased to 63% (±0.06) of the offspring. Using a two-tailed statistical test, this difference was not significant at the 5% level (GLM with binomial error; *F*_1,18_ = 0.6221; *P* = 0.058; Fig. 5). Arguably the use of a one-tailed statistical test is appropriate here because the prediction is directional (inbreeding and exposure should reduce reproductive success), in which case these results would be statistically significant (*P* = 0.029). *Post hoc* analysis revealed that a sample size of 75 (versus *n* = 20 used here) would have been great enough to achieve significance using a two-tailed test, assuming that the observed trend was maintained.

#### Quantification of 11-kt in male plasma

**Figure 6:**
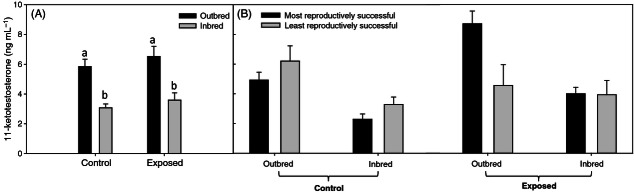
Comparison of plasma 11-kt concentrations in male zebrafish from Experiment II between (A) control and exposed, outbred and inbred fish (*n* = 20), and (B) the most and least reproductively successful male zebrafish, split by treatment group and degree of inbreeding (*n* = 9). Reproductive success was determined by the number of offspring sired by each male within each spawning group. Different letters denote significant differences between treatment groups.

Plasma 11-kt concentrations in male fish in Experiment II were as follows: 6.2 ± 0.42 ng mL^−1^ in control outbreds; 3.24 ± 0.23 ng mL^−1^ in control inbreds; 6.29 ± 0.44 ng mL^−1^ in exposed outbreds; and 3.40 ± 0.27 ng mL^−1^ in exposed inbreds. Three samples that recorded below the limit of detection were assigned a value of half of that of the detection limit (0.61 ng mL^−1^). There were no overall differences in 11-kt levels between control and exposed treatment groups (three-way anova with Holm–Sidak multiple comparison procedure, *F*_1,29_ = 3.33, *P* = 0.078, Fig. 6A). However, in both control and clotrimazole exposed males, levels were significantly higher in outbred fish compared with inbred fish (*F*_1,29_ = 19.43, *P* = <0.001, Fig. 6a). There was no statistically significant interaction between treatments and the degree of inbreeding (*F*_1,29_ = 0.01, *P* = 0.92).

Levels of 11-kt were also analysed based on the proportion of embryos sired by each male. In the control group, there were no significant differences in plasma 11-kt concentrations between male fish siring the highest proportion of offspring (the most reproductively successful males; 3.75 ± 0.56 ng mL^−1^), and the male fish siring the lowest proportion of offspring (the least reproductively successful males; 4.66 ± 0.77 ng mL^−1^), irrespective of the degree of inbreeding. However, in the clotrimazole treatment group, the most reproductively successful outbred males had significantly higher plasma 11-kt levels (8.713 ± 0.86 ng mL^−1^) compared to the least successful males (4.559 ± 1.40 ng mL^−1^; three-way anova with Holm–Sidak multiple comparison procedure, *F*_1,29_ = 6.899, *P* = 0.014, Fig. 6B). This trend was not observed in inbred fish, and there was no overall statistically significant interaction between the degree of inbreeding, exposure to clotrimazole and the proportion of offspring sired (*F*_1,29_ = 2.129, *P* = 0.087).

### Inbreeding depression

Inbreeding depression coefficient values (δ) were calculated for key reproductive traits. In Experiment I, where fish in each spawning group were either all inbred or all outbred, estimates of δ values (×100 for ease of comparison) in control and clotrimazole exposed fish were −1.4 and 24.0 for embryo viability, and 9.9 and −0.5 for the number of eggs produced, respectively. Thus, the addition of chemical stress increased inbreeding depression for embryo viability, but had no great effect on egg production. In Experiment II, we calculated inbreeding depression for siring success in control and exposed fish. We found no evidence for reduced siring success when fish were inbred and not exposed to clotrimazole (δ = −12.8: i.e. inbred males were more successful than outbred males), but with exposure, inbreeding depression in siring success was recorded (δ = 21.3). Thus, as with embryo viability, additional stress caused by clotrimazole exposure amplified the effects of inbreeding on a key male fitness component. Plasma 11-kt concentrations measured in fish in Experiment II showed δ values of 48.0 and 43.5 for control and exposed fish, respectively. Therefore, for this parameter, we found substantial inbreeding, which was not amplified by environmental stress.

## Discussion

In this study, we found that inbreeding depression in a range of critical fitness determinants only became apparent when fish were additionally stressed by exposure to an EDC, the fungicide clotrimazole. Overall, we found that the additional stress caused by clotrimazole exposure increased inbreeding depression in half of the traits we assessed. Thus, our results are consistent with an increasing body of evidence suggesting stress can at least sometimes amplify inbreeding depression. In a meta-analysis of published experimental data, stress significantly increased inbreeding depression in 48% of cases reviewed (Armbruster and Reed 2005). Inbred individuals are expected to be less fit than outbred individuals because of increased homozygosity at genetic loci influencing fitness (Slate et al. 2000), and populations with low genetic variation appear less able to adapt to changes in environmental conditions, including exposure to different physical and chemical stressors (Brown et al. 2009). However, in line with the variability observed in our results, the impact of additional stress on inbreeding depression appears to vary across populations (Keller and Waller 2002) and trait types (Roff 1997).

To our knowledge, this is the first study examining the combined effects of inbreeding and exposure to an EDC in fish. Previous studies have shown inbreeding in fish can cause significant perturbations in life-history traits. For example, inbreeding has been shown to increase fry abnormalities, as well as reduce feeding efficiency, growth rate and fry survival in rainbow trout (*Oncorhynchus mykiss,* Walbaum) (Aulstad and Kittelsen 1971; Kincaid 1976; Gallardo and Neira 2005) and inbreeding lowers fertilization rates and egg hatching success in three spined sticklebacks (*Gasterosteus aculeatus,* Linnaeus) (Frommen et al. 2008). Similarly, in zebrafish, inbreeding leads to a significant reduction in fry survival and growth to 30 days post-hatch (Mrakovčič and Haley 1979).

Our results show that embryo viability significantly declines under the combined effects of exposure and inbreeding. However, inbreeding alone (theoretical *F* = 0.25) had no effect on reproductive output. Lack of inbreeding effects on egg production is consistent with inbreeding studies using other strains of zebrafish (Piron 1978; Mrakovčič and Haley 1979). Furthermore, the lack of effects of clotrimazole exposure on fecundity compare well with those of Ankley et al. (2007) following exposure of fathead minnow (*Pimephales promelas,* Rafinesque) to ketoconazole (7 μg L^−1^), another azole fungicide, and with other studies indicating an apparent resilience of zebrafish egg output to perturbations through chemical exposure. The decline in egg production over the two study periods is likely related to the higher levels and frequency of aggressive behaviours typical of fish maintained in small breeding groups (Paull et al. 2008), which may impact on the ability to maintain high rates of egg production over prolonged periods.

Paternity analysis in the non-exposed spawning triads revealed inbreeding alone (one generation of full-sibling inbreeding) did not affect male reproductive success. This is surprising given the apparent sensitivity of male fertility to inbreeding in other species (Okada et al. 2011). However, in spawning triads previously exposed to clotrimazole, there was a strong trend for outbred males to have superior siring success compared to inbred males. This apparent difference in paternity share could partly explain why embryo viability was decreased in the inbred spawning triads in Experiment I but not in Experiment II (where inbred females were teamed with both inbred and outbred males). The magnitude of inbreeding depression for siring success following exposure was about 21%, suggesting the additional stress caused by the fungicide was significant for this key male fitness component. This estimate of inbreeding depression is similar to those reported for other male life-history traits [e.g. 19% for male fertility in *Drosophila simulans* (Okada et al. 2011) and 18% for longevity in *D. melanogaster* (Hughes 1995)]. Inbreeding depression has also been found to affect other aspects of male reproduction, particularly male attractiveness, including copulation latency in *D. simulans* (Okada et al. 2011), sperm number and ornamental traits in guppies *Poecilia reticulate* (Peters) (Zajitschek and Brooks 2010), and the courtship calls of male crickets (Drayton et al. 2010).

The reduced (50%) plasma 11-kt concentrations seen in inbred fish cannot be attributed to size, as male zebrafish within each spawning triad were sized matched before the breeding studies commenced. We found substantial inbreeding depression in this trait, which was not affected by exposure to clotrimazole. High 11-kt levels in male fish have been correlated with behavioural dominance (Borg 1994) and increased reproductive success (Coe et al. 2008). We found no difference in plasma 11-kt concentrations between the most and least reproductively successful males in the control fish. However, in this study, outbred exposed males had high 11-kt levels and increased reproductive success, corresponding with an increase in paternity share of offspring. Although disruption to plasma 11-kt levels is consistent with the mode of action of clotrimazole (aromatase inhibition), it is unclear why this response would differ between inbred and outbred fish.

On balance, our data suggest inbreeding combined with clotrimazole exposure at a concentration marginally exceeding maximum predicted environmental concentrations [PEC_local_ = 0.2 μg L^−1^ (OSPAR 2005)] can reduce the reproductive competitiveness of male zebrafish. However, a larger scale study (adopting a similar competitive breeding design) would be needed to demonstrate this unequivocally. An alternative to this might be to simulate increased competition, for example, between four males as opposed to only two, to better discern chemical effects, as has been shown for 17α-ethinyloestradiol (Coe et al. 2009). In addition, maintaining chemical exposure during the breeding trials could allow a direct assessment of the chronic effects of clotrimazole exposure and inbreeding on adult spawning, as well as combined acute toxicity effects on embryonic life stages.

In conclusion, our data have shown that inbreeding can impact on fitness-related reproductive traits. However, not all traits are affected equally, and in some cases, inbreeding depression only becomes apparent with the additional stress of chemical exposure. This is one of the first studies reporting that effects of exposure to an EDC on reproductive success can differ between outbred and inbred animal strains. Consequently, EDCs may potentially affect inbred wild populations differently to outbred wild populations, and this is likely to be important when considering how populations might respond evolutionary to pollutants. Our data indicate the importance of better understanding interactions between pollutants and inbreeding. This is particularly pertinent when considering that both inbreeding and adaptive selection because of toxicant exposure may impact on the maintenance of genetic diversity and potential for future evolutionary change in wild populations, as well as the response of laboratory-maintained fish in ecotoxicology tests. Furthermore, this study also provides useful insights into the environmental relevance of using laboratory strains in ecotoxicology when addressing the consequences of exposure to chemical pollutants on the fitness of wild populations. An option for future ecotoxicology testing studies may be to measure genetic diversity of the animal models used. However, this may not be practical in many cases. When considering that genetically impoverished populations are generally more sensitive to the effects of chemical exposure, including results from the current study, inbred laboratory strains may provide adequate protection for the wild populations they are designed to protect. Additional approaches that may benefit the interpretation of ecotoxicology data in this respect include reporting of pedigree records, routinely checking the sensitivity of laboratory strains using standard toxicants, or the use of positive controls in standardized tests.

## Data archiving statement

Data for this study are available at Dryad – doi:10.5061/dryad.6h822.

## Supporting Information

**Table S1.** Experimental Conditions.

**Table S2.** Fish Size.
