# Supplementary material for: Interactive effects of inbreeding and endocrine disruption on reproduction in a model laboratory fish
Source: Evol Appl. 2012 Aug 30;6(2):279–89. doi: 10.1111/j.1752-4571.2012.00288.x (PMC3689353; doi:10.1111/j.1752-4571.2012.00288.x)
Supplement: Supplementary file 1 [file eva0006-0279-SD1.pdf]

Electronic Supplementary Material

Table S1 Experimental Conditions

Details of experimental set up conditions and fish husbandry regime

Parameter	Experimental Conditions
**Water Temperature**	28 ± 1°C
**Dissolved Oxygen**	≥ 60 % saturation
**pH**	6.5 – 8.5
**Photoperiod**	12h:12h light:dark<br>07:00 – 19:00 with an artificial dawn/dusk transition of 20 minutes
**Feeding Regime**	2x/day<br>• freshly hatched *Artemia nauplii* (4 % body weight per day; ZM Premium Grade *Artemia*, ZM, Hampshire, UK)<br>• pelleted food (300 pellet, Special Diets Service, Witham, UK)
**Aquaria**	450 x 350 x 300 mm in dimension<br>12L working volume and a flow-through of 6 tank volume changes per day.<br>Contained a central spawning site, consisting of a metal tray (150 x 100 x 20 mm) filled with glass marbles and an artificial weed placed in the middle.<br>Opaque dividers were placed between aquaria to prevent visual interactions between neighbouring spawning groups.
**Analysis of clotrimazole concentrations (µg/L) (for more information, refer to Brown *et al.*, 2011)**	Chemical analysis was undertaken on Exposure Days -1, 1, 5, 8, 14, 21, 30, 41, 54, 68, 82 and 96, in control and exposure tanks.<br>Control (0 µg L^{-1} nominal)<br>• Arithmetic mean 2.98 µg L^{-1}<br>• Geometric mean 2.87 µg L^{-1}<br>• 95 % CI 0.57<br>• Limit of Detection 0.5 µg L^{-1}<br>Clotrimazole (5µg L^{-1} nominal)<br>• Arithmetic mean 0 µg L^{-1}<br>• Geometric mean 0 µg L^{-1}<br>• 95 % CI 0.1<br>• Limit of Detection 0.5 µg L^{-1}
**Fish Health and General Condition**	All fish responded well to feeding and were checked twice daily for any visible signs of ill health or abnormal swimming behaviour. Visual observations to confirm normality and apparent health of internal organs were made during necropsy.

Table S2 Fish Size

Standard length and wet weight for zebrafish in each treatment group

Experiment I: average size difference between male fish in spawning groups 0.017 ± 0.002g [mean ± standard error]; average size difference between female and male fish in spawning groups 0.092 ± 0.010g

Experiment II: average size difference between male fish in spawning groups 0.014 ± 0.003g; average size difference between female and male fish in spawning groups 0.090 ± 0.007g

**Fish Size**		**Control**		**Clotrimazole Exposed (2.9 µg L^{-1})**	
		**Outbred**	**Inbred**	**Outbred**	**Inbred**
**Female**	Weight (g)	0.321 ± 0.02	0.315 ± 0.03	0.315 ± 0.02	0.331 ± 0.02
	Standard Length (mm)	25.98 ± 8.2	26.54 ± 8.4	26.31 ± 8.3	26.31 ± 8.3
**Male**	Weight (g)	0.252 ± 0.01	0.241 ± 0.01	0.242 ± 0.01	0.247 ± 0.01
	Standard Length (mm)	25.73 ± 5.8	25.72 ± 5.8	25.88 ± 5.8	25.19 ± 5.6
